# Management of Small Renal Masses

**DOI:** 10.1155/2008/606401

**Published:** 2009-03-29

**Authors:** Jose Rubio Briones, F. Algaba

**Affiliations:** ^1^Urology Department, Valencia Institute of Oncology, C/ Prof. Beltrán Báguena 8, 46009 Valencia, Spain; ^2^Department of Pathology, Fundación Puigvert, C/Cartagena 340, 08025 Barcelona, Spain

When we started to plan this special issue, we were under the thought that we are facing more and more cases of small renal masses in our daily work as urologists and pathologists. This common fact nowadays will probably increase in the near future as radiological studies are more frequently ordered and fortunately we face an increase in longevity, and also as people can get to detect their renal masses before they really arrive to the classic lumbar pain/haematuria/lumbo-abdominal mass symptoms.

First of all, strict definition of small renal mass is lacking; most of the authors consider 4 cm as cut-off, imported from the classical one regarding partial surgery of the kidney and TNM classification; but we all know that these concepts are changing and probably will need to be taken into consideration.

Been sure the increase in detection, we have to precise the different needs of radiological explorations to characterize a small renal mass; is sonography, CT, and MRI necessary for all patients? We are still lacking to differentiate from a standard radiological approach benign and malignant small renal masses. What is the role of percutaneous biopsies in these cases? These (and others) are questions that urologists do not answer uniformly. Economical issues are also important in a public medical system.

When we move to therapeutic aspects, things are even more unresolved. There is an increasing number of small renal masses managed under a strict watchful waiting policy but this is not plausible for all cases. Limits of age and growth rate have been argued again for this approach and most of the times, at least in our country, people are not happy knowing they could harbor a renal cancer been just “observed”.

Regarding active treatment, first radical nephrectomy and lastly open partial nephrectomy have been the gold standard approaches. In fact, main guidelines consider the second the treatment of choice for small renal masses nowadays, having shown the same oncological control compared to radical surgery. During the last decade, laparoscopic partial nephrectomy has emerged with comparable oncological results, adding better cosmetical and perioperative recovery data. The main drawback of laparoscopic partial nephrectomy is its difficulty, being just feasible in experienced centers with high volume of patients.

In the last five years, different nonablative techniques have appeared to compete with partial (open or laparoscopic) nephrectomy aiming to achieve same oncological control, testing percutaneous approach, reducing complication rates, and improving recovery, what have been called minimally invasive treatments. As time goes by, these techniques have failed to demonstrate good and reproducible results in any prospective trial for the percutaneous approach, but this and the laparoscopic approach are increasing in number worldwide, mainly radiofrequency and cryotherapy for small renal masses. Follow-up will tell us if they achieve same cancer control, but preliminary results show acceptable results for cryotherapy and are questionable for radiofrequency.

Our aims are to summarize distinct aspects of the management of small renal masses nowadays, focusing on its epidemiology, pathological aspects, prognosis, and mostly the different treatment strategies.

In the first three manuscripts, the authors try to concrete the clinical problem of small renal masses nowadays, focusing on multifocality and other prognostic factors that could guide their management. Two papers more analyze the familial syndromes involved with small renal masses and the possible genetic counselling we should offer the relatives of patients with these tumors.

The next block studies the different radiological aspects of small renal masses, both in the preoperative scenario and then after treatment, where many doubts about local recurrence need to be clarified by radiologists.

There is an interesting and vast review about the physiopathology of renal ischemia, a crucial point in renal partial surgery. The reader will find the limits of it and the research ongoing in such an “unknown” field.

In the therapeutic block, there are two nice reviews about watchful waiting policy analyzing fresh data. Then, open partial nephrectomy will be reviewed and presented with a comparative intent to laparoscopic partial nephrectomy in 3 papers, and reviews on nonablative techniques will be discussed in two papers more.

Finally, two papers analyze the problems that pathologist face in front of these many-times small renal masses.

We hope that this special issue will answer some of the reader doubts about the management of small renal masses, knowing that the next and near future will offer us much more data that can change our actual point of view.

*Jose Rubio Briones* *F. Algaba*

